# Aerobic conditioning alters the satellite cell and ribosome response to acute eccentric contractions in young men and women

**DOI:** 10.1152/ajpcell.00418.2022

**Published:** 2022-10-24

**Authors:** Alex Brown, Aaron C. Q. Thomas, Aidan A. Hatt, Chris McGlory, Stuart M. Phillips, Dinesh Kumbhare, Gianni Parise, Sophie Joanisse

**Affiliations:** ^1^Exercise Metabolism Research Group, Department of Kinesiology, McMaster University, Hamilton, Ontario, Canada; ^2^School of Kinesiology and Health Studies, Queen’s University, Kingston, Ontario, Canada; ^3^Department of Medicine, Queen’s University, Kingston, Ontario, Canada; ^4^Toronto Rehabilitation Institute, Toronto, Ontario, Canada; ^5^Department of Sport and Exercise Sciences, Musculoskeletal Science and Sport Medicine Research Centre, Institute of Sport, Manchester Metropolitan University, Manchester, United Kingdom

**Keywords:** ribosomes, satellite cells, skeletal muscle, translation, translational capacity

## Abstract

Satellite cells (SCs) and ribosomes are key determinants of the skeletal muscle adaptive response. Both are thought to increase acutely after resistance exercise and chronically with resistance training. However, the acute SC and ribosome exercise response with prior aerobic conditioning is unknown. Fourteen young men and women underwent 6 wk of single-legged aerobic conditioning followed by an acute bout of 300 eccentric contractions on each leg. Muscle biopsies were taken from the vastus lateralis of the aerobically conditioned (AC) and the control (CTL) legs before (Pre), 24 (24 h), and 48 (48 h) h post-contractions. Pre-eccentric contractions, 45S pre-rRNA and 5.8S internal transcribed spacer (ITS) expression were lower in the AC leg compared with the CTL leg. SC content (PAX7^+^ cells/100 fibers) in type I and mixed fibers showed a main effect of condition, where values were greater in the AC leg compared with the CTL. A main effect of condition for *Pax7* and *MyoD1* mRNA expression was observed where expression was greater in the AC leg compared with the CTL. AC had greater RNA concentration and mRNA expression of *Ubf* and *Tif-1a* compared with CTL. Only the AC leg increased (Pre-24h) 45S pre-rRNA, 5.8S ITS, and 28S ITS following eccentric contractions. We discovered that aerobic conditioning increased type-I SC abundance and the acute increase in ribosome content following eccentric contractions.

## Graphical abstract

**Figure:**
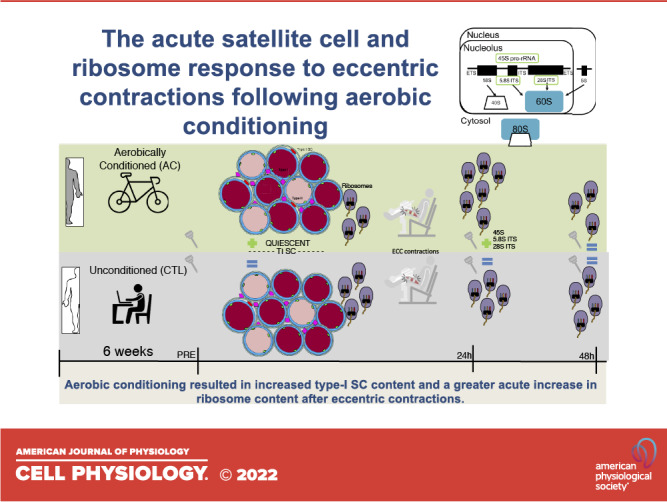

## INTRODUCTION

In humans, eccentric contractions lead to skeletal muscle damage, resulting in the subsequent activation of cellular processes to support repair (1, 2). Muscle-specific stem cells, commonly referred to as satellite cells (SCs), are particularly important for skeletal muscle repair (3–5). Following various stimuli, such as exercise or damage-inducing eccentric contractions, SCs are activated, proliferate and either fuse to existing myofibers to support repair and remodeling or return to quiescence to replenish the SC pool (4–7).

We have previously reported that young individuals with greater skeletal muscle capillarization showed an augmented SC expansion/activation following a single bout of eccentric contractions, resulting in an accelerated recovery of muscle function (8). Therefore, aerobic conditioning (a well-known stimulus to induce skeletal muscle capillarization) preceding an acute damaging stimulus may heighten the SC response and support muscle adaptation and repair (9, 10).

Ribosomes play a key role in protein translation (11–13) and recent work in rodents has demonstrated that SCs may supply certain ribosomal proteins to muscle fibers to support adaptation (14). Following an acute bout of resistance exercise, ribosome content increases to support the synthesis of proteins involved in cellular remodeling (15–18) and muscle contractions (19–21). Increases in ribosome content likely precede muscle protein synthesis, which is stimulated following aerobic (22, 23) and resistance exercise (24, 25). In addition, damage-inducing eccentric contractions increase the expression of genes associated with the regulation of muscle protein synthesis (2). Although ribosomes are essential for regulating protein translation, changes in ribosome content following an acute bout of eccentric contractions have been measured in rodents (26, 27) but not in humans.

The purpose of this study was to determine the impact of aerobic conditioning on the acute SC and ribosome response to eccentric contractions. We hypothesized that ribosome content would increase following acute eccentric contractions and that aerobic conditioning would augment both the SC and ribosome response to eccentric damage.

## MATERIALS AND METHODS

### Ethics Approval

Participants were informed about the nature and risks of the study and gave written consent before enrollment. This study was approved by the Hamilton Health Sciences Integrated Research Ethics Board (HiREB No. 3885) and conformed to the guidelines outlined in the Declaration of Helsinki.

### Participants

Baseline participant characteristics have previously been described by Thomas et al. (28) and are summarized in Table 1.

**Table 1.** Participant characteristics

Characteristic	Males (*n* = 8)	Females (*n* = 6)	Overall (*n* = 14)	RNA Analyses (*n* = 11)
Age, yr	21.0 ± 1.7	21.0 ± 1.5	21.1 ± 1.6	21.3 ± 1.6
Body mass, kg	82.2 ± 15.5*	60.0 ± 9.4	74.1 ± 17.6	75.2 ± 20.6
BMI, kg/m^2^	27.3 ± 4.8	22.9 ± 2.2	25.4 ± 4.5	25.8 ± 5.2
V̇o_2_ relative, mL/min/kg	42.3 ± 6.9	34.8 ± 4.6	39.1 ± 7.1	37.0 ± 5.8

Values are means ± SD. Independent *t* test, *significant difference between males and females (*P* < 0.05). No difference between the “Overall” and “RNA Analyses” groups. BMI, body mass index; V̇o_2_, peak oxygen consumption.

### Study Design

Participants underwent 6 wk of single-legged aerobic conditioning on a randomized leg (aerobically conditioned, “AC”) where the other acted as a nonconditioned control (CTL; 28). Participants underwent resting (Pre) skeletal muscle biopsies from the vastus lateralis of both the AC and CTL legs according to Tarnopolsky et al. (29) at least 1 wk following the last AC bout. Participants then underwent 300 isokinetic, eccentric contractions of the quadriceps muscles at 180°/s using a Biodex dynamometer (Biodex-System 4, Biodex Medical Systems, Shirley, NY) with each leg, a protocol used frequently in our laboratory to elicit skeletal muscle damage (8, 30–32). Participants returned to the laboratory 24 and 48 h following eccentric contractions and underwent biopsies from both the CTL and AC legs. Samples were either mounted in Optimal Cutting Temperature (OCT) compound and frozen in precooled isopentane or frozen in liquid nitrogen and stored at –80°C.

### Immunohistochemical Analyses

Immunofluorescent staining for fiber-specific SC content (PAX7^+^ cells) and activation status (quiescent PAX7^+^/MYOD^−^, activated PAX7^+^/MYOD^+^, and differentiating PAX7^−^/MYOD^+^) are described previously (8, 33–36) and expressed per 100 fibers. All staining procedures were verified for specificity using negative controls for primary (primary only) and secondary (secondary only) antibodies. For quantification, PAX7 (anti-PAX7 Mouse, DHSB, neat; Alexa Fluor 594 goat anti-mouse, 1:500) and/or MYOD (anti-MYOD 5.8 A Mouse, DAKO, 1:100; goat anti-mouse biotin, 1:200, and streptavidin 488, 1:200) was overlayed with DAPI (Sigma-Aldrich, 1:20,000) and examined with laminin (anti-Laminin Rabbit, Abcam ab11575, 1:500; Alexa Fluor 647 goat anti-rabbit 1:500) or wheat germ agglutinin (WGA; Wheat Germ Agglutinin, Invitrogen W32466, 1:200) to determine the appropriate SC location, myosin heavy chain I (anti-MHCI Mouse, DHSB A4.951, neat; Alexa Fluor 488 goat anti-mouse, 1:500) and II (anti-MHCII Rabbit, Abcam ab51263, 1:1,000; Alexa Fluor 647 goat anti-rabbit, 1:500) to determine fiber type-specific associations and expressed per 100 fibers. Images were taken on a Nikon Eclipse Ti Microscope (Nikon Instruments) with a high-resolution Photometrics CoolSNAP HQ2 fluorescent camera (Nikon Instruments, Melville, NY) at a ×20 objective. Analyses were performed in a blinded fashion.

### RNA Isolation and Reverse Transcription

RNA was isolated from muscle homogenate using the TRIzol and reverse-transcribed using the high-capacity cDNA reverse transcription kit (Applied Biosystems, Cat. No. 4368814) according to the manufacturer’s protocol and stored at −20°C until subsequent analysis. Samples from three participants were excluded due to low RNA concentration yield (see Table 1).

### Quantitative Real-Time PCR

Quantitative real-time PCR (RT-qPCR) reactions were run using 10 ng cDNA in a QuantStudio 5—384-well block (Applied Biosystems, Thermo Fisher Scientific) RT qPCR machine. Primer sequences (5′-3′ forward, reverse; concentration) for *Gapdh* ( CCACCCATGGCAAATTC, TGGGATTTCCATTGATGACAA; 15 µM), *Cyclin D1* ( GCTGCGAAGTGGAAACCATC, CCTCCTTCTGCACACATTTGAA; 15 µM), *Ubf* ( CCTGGGGAAGCAGTGGTCTC, CCCTCCTCACTGATGTTCAGC; 10 µM), *Tif-1a* ( GTTCGGTTTGGTGGAACTGTG, TCTGGTCATCCTTTATGTCTGG; 10 µM), *Polr-1b* ( GCTACTGGGAATCTGCGTTCT, CAGCGGAAATGGGAGAGGTA; 10 µM), 5.8S rRNA ( ACTCTTAGCGGTGGATCACTC, GACGCTCAGACAGGCGTAG; 10 µM), 18S rRNA ( TGGCTCAGCGTGTGCCTAC, ACAAAGGGCAGGGACTTAATC; 10 µM), 28S rRNA ( ACCTGGCGCTAAACCATTC, GTGTCGAGGGCTGACTTTC; 10 µM), 5.8S ITS ( TCGCCAAATCGACCTCGTAC, AGCTGCGTTCTTCATCGACG; 10 µM), 18S ETS ( GCCCGTCCTCGCGAGGC, TGCATGGCTTAATCTTTGAGAC; 15 µM), and 28S ITS ( CGGCGCGATTCCGTCCGT, GTTCACTCGCCGTTACTGAG; 10 µM), and assays for *Gapdh* (ThermoFisher, Hs00187842_m), *Pax7* (ThermoFisher, Hs00242962_m1), *MyoD1* (ThermoFisher, Hs00159528_m1), *Myf5* (ThermoFisher, Hs00929416_g1), *c-Myc* (ThermoFisher, Hs00153408_m), 45S pre-rRNA (Qiagen, ID PPH82089A-200), and 5S rRNA (ThermoFisher, Hs03682751_gH) were used. Reactions for individual primers and the 45S pre-rRNA assay were run with RT^2^ Sybr Green qPCR Master Mix (Qiagen, No. 330500) and all other assays using Taqman Fast Advanced Master Mix (ThermoFisher, No. 4444556). The housekeeping gene (*Gapdh*) expression was not impacted by the intervention. Samples were normalized to *Gapdh* (ΔC_t_; either respective SYBR or Taqman *Gapdh*) and to Pre eccentric contractions in the CTL leg (ΔΔC_t_).

### Statistical Analyses

Jamovi 1.6.23 was used to run statistical analyses. Outliers were determined using means ± 2 × standard deviation (SD) and removed from analyses. Trend analyses for missing and removed data were used for participants with one or less missing data points. A paired *t* test was used to determine the change (Δ) in peak oxygen consumption (V̇o_2_) peak between CTL and AC following aerobic conditioning. An independent *t* test was used to compare the Total and RNA group characteristics. SC content and activation and gene expression data were analyzed using a two-way repeated measure analysis of variance with factors of time (Pre, 24 h, and 48 h) and condition (CTL and AC), where Tukey’s honest significant difference test was used to analyze multiple post hoc comparisons.

All data are expressed as means ± standard deviation (SD).

## RESULTS

### Participant Characteristics

Due to tissue availability, only 11 participants (*n* = 6 males, *n* = 5 females) were included in the gene expression analyses compared with 14 (*n* = 8 males, *n* = 6 females) in the immunohistochemical analyses (Table 1). Both the “Total (*n* = 14)” and “Gene expression analyses (*n* = 11)” groups had a similar age (21 ± 2 yr), body mass index (BMI; *n* = 14, 25.4 ± 4.7; *n* = 11, 25.8 ± 5.2 kg/m^2^), and ΔV̇o_2_ peak (*n* = 14, 3.9 ± 3.6; *n* = 11, 3.7 ± 3.0 mL/min/kg) from pre-AC to post-AC (*P* > 0.05).

### Satellite Cell Content

A significant time effect was observed for type-I (*P* = 0.000232), type-II (*P* < 0.0001), and mixed fiber (*P* < 0.0001) SC content.

**Figure 1.**
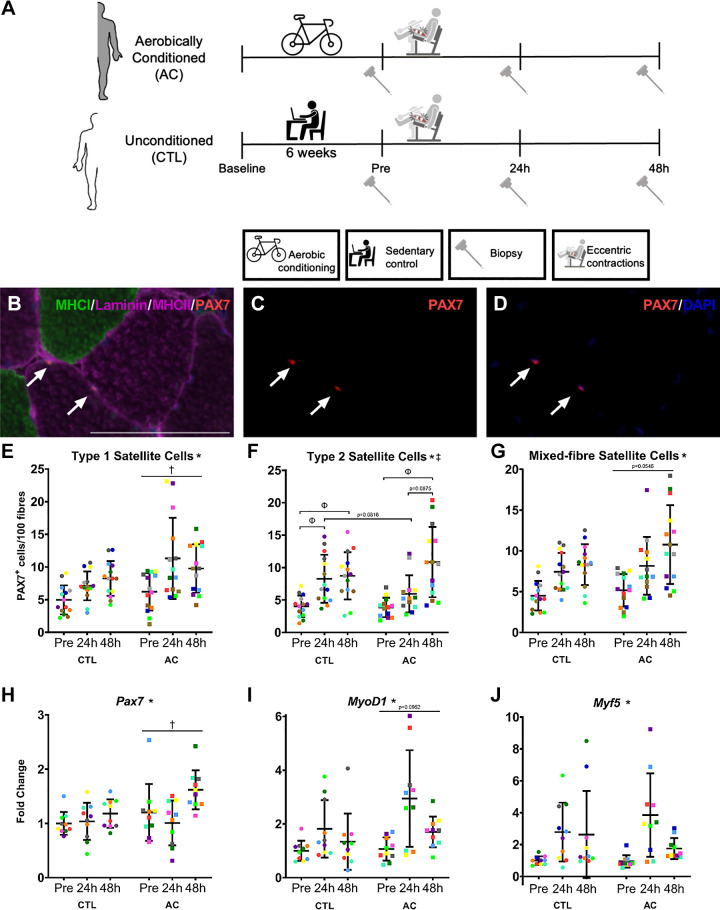
SC content and myogenic gene expression. *A*: schematic of the study design. Representative images of immunofluorescent stains for MHCI, Laminin, MHCII and PAX7 overlayed (*B*), PAX7 (*C*), and PAX7 and DAPI (*D*). The white arrows indicate PAX7^+^/DAPI^+^ cells and the scale bar is 100 μm. SC per 100 fibers for SC located to type-I (*E*), type-II (*F*), and mixed-fibers (*G*; *n* = 14 participants). Myogenic genes *Pax7* (*H*; *n* = 10), *MyoD1* (*I*; *n* = 10), and *Myf5* (*J*; *n* = 10) mRNA expression Pre, 24, and 48 h following eccentric contractions. All values are individual data points for the CTL (•) and AC (■) legs, where each color represents a different participant and is overlayed on means (middle, horizontal line) ± SD (vertical line). Two-way repeated measures of variance, *significant effect of time, †significant effect of condition (AC > CTL), ‡significant time × condition interaction, Φsignificant difference between means (Tukey’s honest significant difference test, *P* < 0.05). AC, aerobically conditioned; CTL, control; MHCI, myosin heavy chain I; SC, satellite cells.

A significant effect of condition was observed for type-I SC content (Fig. 1*D*; PAX7^+^ cells) where the AC leg was greater than the CTL leg (*P* = 0.0184) and tended to have greater mixed-fiber SC content (Fig. 1*F*; *P* = 0.0546).

A significant time × condition interaction was observed for type-II SC content (Fig. 1*E*; *P* = 0.00228), where the CTL leg significantly increased type-II-specific SC content from Pre to 24 h (*P* = 0.00702) and 48 h (*P* = 0.00616) posteccentric contractions. The AC leg increased from Pre to 48 h (*P* = 0.00319) but was not different at 24 h (*P* > 0.05).

No time × condition interactions were observed for type-I or mixed-fiber SC content (*P* > 0.05).

### Myogenic Gene Expression

A significant effect of time was observed for *Pax7* (*P* = 0.0105), *MyoD1* (*P* < 0.0001), and *Myf5* (*P* = 0.00217) mRNA expression.

A significant effect of condition was observed for *Pax7* mRNA expression (Fig. 1*G*; fold-change), where the AC leg was greater than the CTL (*P* = 0.00419) and tended to have greater *MyoD1* (Fig. 1*H*; *P* = 0.0952) but not *Myf5* (Fig. 1*I*; *P* > 0.05) mRNA expression. No time × condition interactions were observed (*P* > 0.05).

### Satellite Cell Activation Status

A significant effect of time was observed for type-I activated (PAX7^+^/MYOD^+^; *P* = 0.00489), type-II quiescent (PAX7^+^/MYOD^−^; *P* = 0.0220), and type-II activated (*P* = 0.00256) SC content.

**Figure 2.**
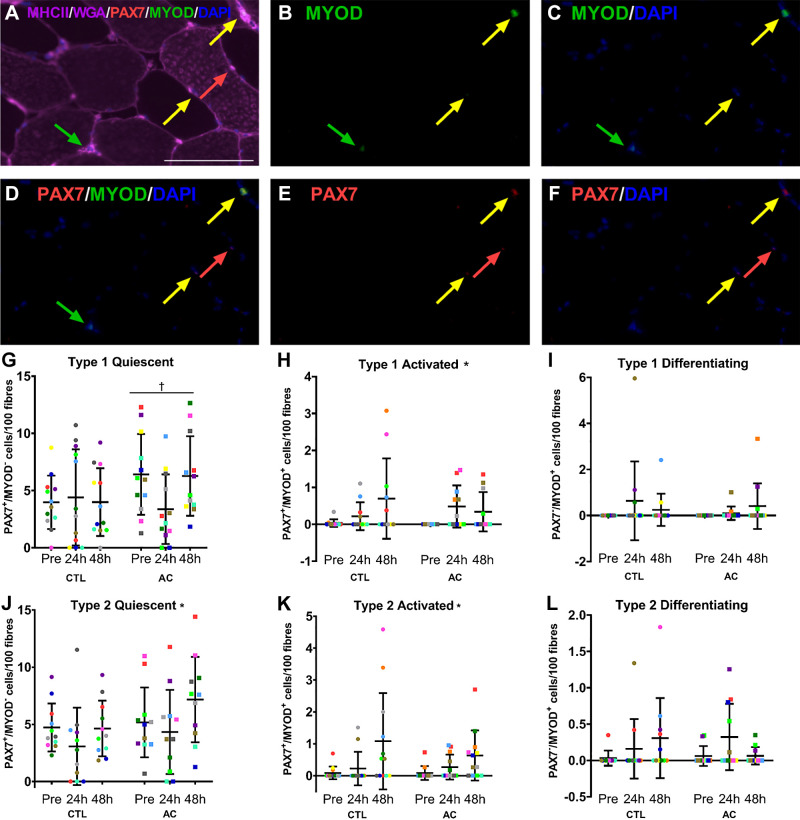
SC activation status. Representative images of immunofluorescent stains for PAX7, MYOD, MHCII and WGA, and DAPI overlayed (*A*), MYOD (*B*), MYOD and DAPI (*C*), PAX7, MYOD, and DAPI (*D*), PAX7 (*E*), and PAX7 and DAPI (*F*). The red arrows indicate PAX7^+^/MYOD^−^ cells, yellow arrows indicate PAX7^+^/MYOD^+^ cells, and green arrows indicate PAX7^−^/MYOD^+^ cells, and the scale bar is 100 μm. Type-I-specific quiescent (PAX7^+^/MYOD^−^; *n* = 12 participants; *G*), activated (PAX7^+^/MYOD^+^; *n* = 11; *H*), and differentiating (PAX7^−^/MYOD^+^; *n* = 12; *I*) SC. Type-II-specific quiescent (*n* = 11; *J*), activated (*n* = 12; *K*), and differentiating (*n* = 11; *L*) SC Pre, 24, and 48 h following eccentric contractions. All values are individual data points for the CTL (•) and AC (■) legs, where each color represents a different participant and is overlayed on means (middle, horizontal line) ± SD (vertical line). Two-way repeated measures of variance, *significant effect of time, †significant effect of condition (AC > CTL). AC, aerobically conditioned; CTL, control; MHCII, myosin heavy chain II; SC, satellite cells; WGA, wheat germ agglutinin.

A significant effect of condition was observed for type-I quiescent SC content (Fig. 2*G*), where the AC leg was greater than the CTL leg (*P* = 0.00427). No differences were observed for activated or differentiating SC content between legs (*P* > 0.05). No time × condition interactions were observed (*P* > 0.05).

### [RNA]

**Figure 3.**
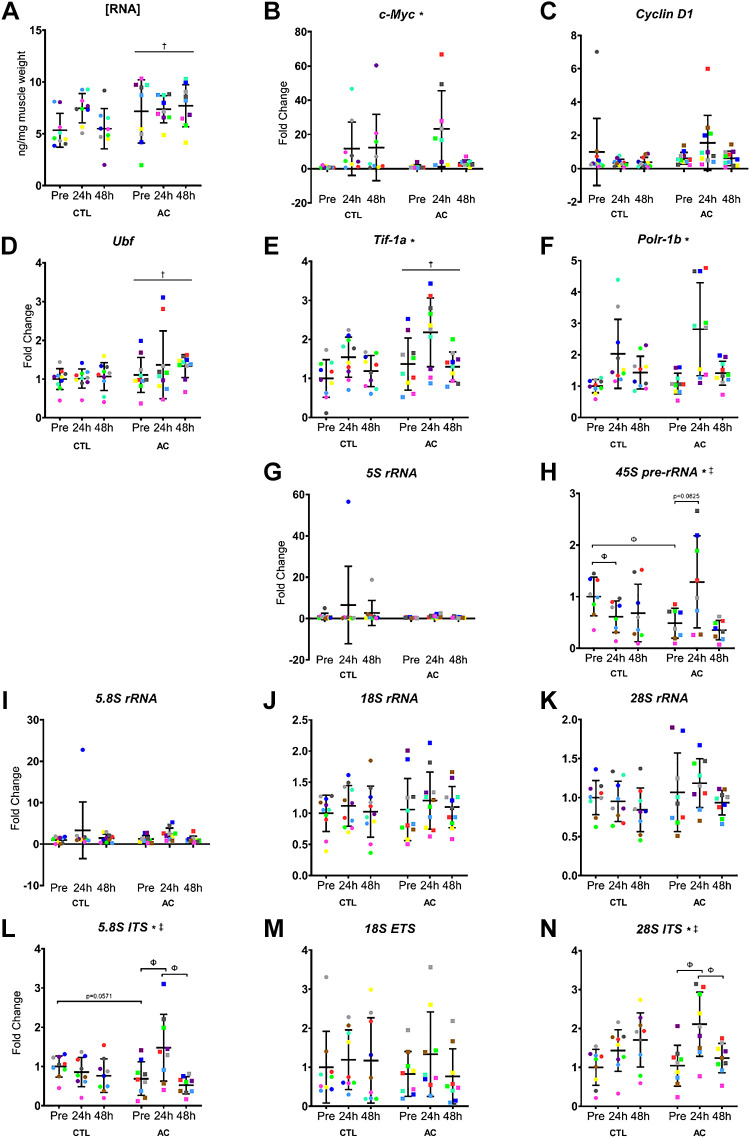
Markers of ribosomal biogenesis and ribosome content. *A*: RNA concentration relative to muscle wet weight (*n* = 9 participants). Ribosomal biogenesis markers *c-Myc* (*n* = 9; *B*), *Cyclin D1* (*n* = 11; *C*), *Ubf* (*n* = 10; *D*), *Tif-1a* (*n* = 10; *E*), and *Polr-1b* (*n* = 10; *F*) mRNA expression, ribosomal RNA markers 5S rRNA (*n* = 9; *G*), 45S pre-rRNA (*n* = 8; *H*), 5.8S rRNA (*n* = 10; *I*), 18S rRNA (*n* = 11; *J*), 28S rRNA (*n* = 9; *K*), 5.8S ITS (*n* = 9; *L*), 18S ETS (*n* = 9; *M*), 28S ITS (*n* = 9; *N*) expression Pre, 24, and 48 h following eccentric contractions. All values are individual data points for CTL (•) and AC (■), where each color represents a different participant and is overlayed on means (middle, horizontal line) ± SD (vertical line). Two-way repeated measures of variance, *significant effect of time (Pre RT > Post RT), †significant effect of condition (AC > CTL), ‡significant time × condition interaction, Φsignificant difference between means (Tukey’s honest significant difference test, *P* < 0.05). AC, aerobically conditioned; CTL, control.

A significant effect of condition was observed for [RNA] (Fig. 3*A*; ng/mg muscle), where the AC leg was greater than the CTL leg (*P* = 0.00982).

### Ribosomal Biogenesis Regulators

A significant effect of time was observed for *c-Myc* (Fig. 3*B*; *P* = 0.0134), *Tif-1a* (Fig. 3*E*; *P* < 0.0001), and *Polr-1b* (Fig. 3*F*; *P* < 0.0001) mRNA expression.

*C-Myc* mRNA expression (fold change) tended to increase from Pre (1.12 ± 1.07) to 48 h after damage (3.31 ± 1.81) in the AC leg (*P* = 0.0733; Fig. 3*B*).

Significant effects of condition were observed for *Ubf* (Fig. 3*D*; *P* = 0.0489) and *Tif-1a* (Fig. 3*E*; *P* = 0.00436) mRNA expression, where the AC leg was greater than the CTL leg. No effects of condition were observed for *Cyclin D1* (Fig. 3*C*) or *Polr-1b* (Fig. 3*F*) mRNA expression (*P* > 0.05). No time × condition interactions were observed (*P* > 0.05).

### Ribosomal RNAs

A significant effect of time (*P* = 0.00392) and time × condition interaction (*P* = 0.0117) was observed for 45S pre-rRNA expression (Fig. 3*H*). The AC leg tended to increase 45S pre-rRNA expression from Pre to 24 h posteccentric contractions (*P* = 0.0825), whereas the CTL leg had significantly greater 45S pre-rRNA expression Pre eccentric contractions compared with the AC leg (*P* = 0.00297) and decreased at 24 h (*P* < 0.0001).

No effects of time, condition, or time × condition interactions were observed for 5S rRNA (Fig. 3*G*), 5.8S rRNA (Fig. 3*I*), 18S rRNA (Fig. 3*J*), or 28S rRNA (Fig. 3*K*) expression (*P* > 0.05).

### Internal and External Transcribed Spacer Regions

Significant effects of time (*P* = 0.00145, *P* = 0.00173) and time × condition interactions (*P* = 00530, *P* = 0.000507) were observed for 5.8S ITS (Fig. 3*L*) and 28S ITS (Fig. 3*N*) expression, respectively. The AC leg significantly increased 5.8S ITS expression from Pre to 24 h (*P* = 0.0347), then decreased from 24 h to 48 h (*P* = 0.0412). The CTL leg tended to have greater 5.8S ITS expression Pre eccentric contractions compared with the AC leg (*P* = 0.0571). The AC leg significantly increased 28S ITS expression from Pre to 24 h (*P* = 0.0151), then decreased from 24 h to 48 h (*P* = 0.0418). No effects of time, condition, or time × condition interactions were observed for 18S ETS expression (Fig. 3*M*; *P* > 0.05).

## DISCUSSION/CONCLUSIONS

We report that the type-I fiber-associated SC content and that the acute increase in ribosome content were greater following acute eccentric contractions preceded by AC compared with the CTL. Nonetheless, no differences between conditions were observed for SC activation, differentiation, or type-II-associated SC expansion. This study is the first to characterize the acute SC and ribosome response with AC and to determine the impact of eccentric contractions on the change in ribosome content in humans.

We have previously demonstrated that individuals with greater skeletal muscle capillarization have a greater SC response to damage-inducing exercise (8) suggesting that muscle capillarization may be a key factor governing SC function. In addition, studies in both humans (33, 34) and mice (37, 38) have demonstrated that aerobic conditioning alters SC dynamics to break quiescence and increase the number of activated SC at rest (humans) and following a damaging stimulus (mice). Work in middle-aged women has also demonstrated that endurance training is able to alter the acute SC response to a bout of resistance exercise (39). The participants in the present study experienced an increase in V̇o_2_ peak and skeletal muscle capillarization following single-legged AC (28), which was associated with an augmented type-I SC content, further supporting the notion that training status and specifically capillary content can impact SC function.

The muscle-damaging protocol that we used in the current study has been used on numerous occasions by our group (8, 30, 31, 35) and others (1, 2, 40). We report an effect of condition for a greater type-I-specific Pax7^+^ and quiescent SC content in the AC leg compared with the CTL. As total type-I PAX7^+^ cells appear similar between legs before eccentric contractions, this may indicate that type-I SC was primed to respond to stimuli as aerobic conditioning primarily targets type-I fibers (39). This finding is consistent with the AC leg having greater mRNA expression of *Pax7* and tending to have a greater mRNA expression of *MyoD1*, but in contrast with a previous study in which middle-aged women completed 12 wk of aerobic training and an increase in type-I SC content was reported (41). However, it is important to note that there were differences in both study populations (young men and women compared with middle-aged women), and an increase in type-I fiber CSA following the aerobic stimulus was reported in middle-aged women, which may explain the increase in type-I associated SC content. Another study in sedentary middle-aged individuals that completed 12 wk of aerobic conditioning also reported an increase in type-I SC content, however, this was also accompanied by an increase in type-I fiber CSA (42). Although the participants in our study did not increase type-I CSA following aerobic conditioning (28), previous work by our laboratory has demonstrated an increase in activated SC following aerobic conditioning, which may act as an anticipatory response for future stimuli (34).

Although type-I and mixed-fiber-specific SC content were greater in the AC leg compared with the CTL, only type-II-specific SC content increased following eccentric contractions. Both the AC and CTL legs increased type-II SC content to a similar extent, however, the CTL leg increased total PAX7^+^ cells at 24 h, whereas the AC leg showed delayed PAX7^+^ cell accumulation, peaking at 48 h. The number of activated (PAX7^+^/MYOD^+^) SC increased in both type-I and -II fibers following eccentric contractions with no difference between conditions. Therefore, AC augmented type-I-specific SC content and appeared to delay the acute increase in type-II SC content, but did not appear to influence the type-I or -II activation or differentiation status.

Ribosome biogenesis increases acutely following resistance exercise to synthesize new ribosome complexes; however, following resistance training, the acute increase in ribosome content following a bout of resistance exercise may be blunted (15–18). Aerobic conditioning resulted in greater expression of several ribosome-related genes. Expression of ribosomal biogenesis regulators upstream binding factor (*Ubf*) and transcription intermediary factor 1 A (*Tif-1a*) were greater in the AC leg. *C-Myc*, the master regulator of ribosomal biogenesis (43), has previously been demonstrated to peak at 8 h and return to baseline at 24 h following an acute bout of exercise (44). In the current study, we report an increase in *C-Myc* 48 h posteccentric contractions in the AC leg. The differences in this increase could be due to the differences in exercise stimulus, where perhaps a more damaging stimulus could delay the spike in *c-Myc* expression. These observations (alongside the other ribosome biogenesis markers) indicate a greater capacity for the AC leg to increase ribosome content following eccentric contractions. Ribosomal RNAs did not increase following eccentric contractions and were similar between conditions, likely due to the high degree of interindividual variability previously observed in their expression (44, 45). However, 45S pre-rRNA, 5.8S ITS, and 28S ITS increased expression 24 h posteccentric contractions in the AC leg and returned to baseline after 48 h, which aligns with previous work (44). The increase and subsequent decrease in 45S pre-rRNA and ITS expression suggests that ribosome content increases following eccentric contractions and that the increase is greater in the AC leg.

The impacts of exercise training on the acute changes in ribosome content are not well understood. The only studies to measure acute changes in ribosome content following resistance training reported either no change or an increase (15) and no change or a decrease (46) in markers of ribosomal biogenesis. Our study is the first to measure the change in ribosome content following an acute bout of exercise (any type) after a period of AC and the first to measure these acute changes beyond the 1 h-acute timepoint. It appears that AC augments the acute increase in ribosome content following eccentric contractions and therefore, suggests that AC may “prime” ribosomes to respond to a novel stimulus.

We discovered that AC augments type-I and mixed-fiber SC content and the acute increase in ribosome content following eccentric contractions. The greater SC content and markers of ribosome biogenesis, and acute increases in ribosome content following eccentric contractions in the AC leg indicate a more efficient transcription and translational control in exercise-accustomed muscle to better support repair and adaptation to damaging stimuli. Future work should measure protein synthesis and specific subfractions (i.e., myofibrillar, sarcoplasmic, and mitochondrial) in response to eccentric damage and markers of translational efficiency, another important determinant in protein synthesis (13, 43).

## GRANTS

This study was supported by a Discovery grant from the Natural Sciences and Engineering Research Council of Canada (RGPIN-2016-05633) awarded to G.P. A.B. was supported by an Ontario Graduate Scholarship. A.C.Q.T. was supported by a Canadian Graduate Scholarship (Masters) from the Natural Sciences and Engineering Research Council of Canada.

## DISCLOSURES

Dr. Phillips reports grants from US National Dairy Council, Dairy Farmers of Canada, Roquette Freres, National Science and Engineering Research Council, and Canadian Institutes for Health Research during the conduct of the study; personal fees from US National Dairy Council, non-financial support from Enhanced Recovery, outside the submitted work; In addition, Dr. Phillips has a patent Canadian 3052324 issued to Exerkine, and a patent US 20200230197 pending to Exerkine but reports no financial gains. None of the other authors has any conflicts of interest, financial or otherwise, to disclose.

## AUTHOR CONTRIBUTIONS

A.C.Q.T., S.M.P., D.K., G.P., and S.J. conceived and designed research; A.B., A.C.Q.T., A.A.H., C.M., and S.J. performed experiments; A.B., A.A.H., and S.J. analyzed data; A.B., S.M.P., G.P., and S.J. interpreted results of experiments; A.B. and S.J. prepared figures; A.B. and S.J. drafted manuscript; A.B., A.C.Q.T., C.M., D.K., G.P., and S.J. edited and revised manuscript; A.B., A.C.Q.T., A.A.H., C.M., S.M.P., D.K., G.P., and S.J. approved final version of manuscript.
